# Supplementary material for: The Function of Mitochondrial Calcium Uniporter at the Whole-Cell and Single Mitochondrion Levels in WT, MICU1 KO, and MICU2 KO Cells
Source: Cells. 2020 Jun 22;9(6):1520. doi: 10.3390/cells9061520 (PMC7349584; doi:10.3390/cells9061520)
Supplement: Supplementary file 1 [file cells-09-01520-s001.pdf]

Supplementary Information Text:

The Function of Mitochondrial Calcium Uniporter at the Whole-Cell and Single Mitochondrion Levels in WT, MICU1 KO, and MICU2 KO Cells

Syed Islamuddin Shah and Ghanim Ullah

Bioenergetics model

To investigate how Ca^{2+} uptake affects mitochondrial bioenergetics in WT, MICU1 KO, and MICU2 KO cells, we adopt the equations used by Wacquier et al. [1]. The mitochondrial NADH ($[\mathrm{NADH}]_\mathrm{M}$), ADP ($[\mathrm{ADP}]_\mathrm{M}$), cytosolic ADP ($[\mathrm{ADP}]_\mathrm{C}$) concentrations, and mitochondrial membrane potential ($\Delta\psi$) are modeled by four rate equations and three conservation equations. Most of these equations were adopted or modified from Refs. [2-5]. In the following, we briefly describe these equations and refer the interested reader to Ref. [1] for more details. The values and meaning of various parameters used are given in Table S1.

Evolution equations

$[\mathrm{NADH}]_\mathrm{M}$), $[\mathrm{ADP}]_\mathrm{M}$, $[\mathrm{ADP}]_\mathrm{C}$, and $\Delta\psi$ are given by the following rate equations.

$$\frac{d[\mathrm{NADH}]_M}{dt} = V_{PDH} - V_O + V_{AGC}, \qquad (S1)$$

$$\frac{d[\mathrm{ADP}]_\mathrm{M}}{dt} = V_{ANT} - V_{FIFO}, \qquad (S2)$$

$$\frac{d[\mathrm{ADP}]_\mathrm{C}}{dt} = V_{HYD} - \delta V_{ANT}, \qquad (S3)$$

$$\frac{d\Delta\Psi}{dt} = \frac{\left(a_1 V_O - a_2 V_{FIFO} - V_{ANT} - V_{H,leak} - V_{NaCa} - 2V_{MCU} - J_{AGC}\right)}{C_{mito}}. \qquad (S4)$$

Where δ, a_1, a_2, and C_{mito} is mitochondrial to cytosolic volume ratio, scaling factor between NADH consumption and change in membrane potential, scaling factor between ATP production by ATP synthesis by F_1F_O-ATPase and change in membrane potential, and mitochondrial inner membrane capacitance respectively. Various fluxes used in Eqs. S1-S4 are given below.

Conservation equations

Conservation of total $[NADH]_\mathrm{M}$, di- and tri-phosphorylated adenine nucleotides in mitochondria, and of di- and tri-phosphorylated adenine nucleotides in the cytosol is given by the following equations.

$$[\mathrm{NADH}]_\mathrm{M} + [\mathrm{NAD}^+]_\mathrm{M} = [\mathrm{NAD}]_\mathrm{M}^\mathrm{TOT}, \qquad (S5)$$

$$[\mathrm{ADP}]_\mathrm{M} + [\mathrm{ATP}]_\mathrm{M} = [\mathrm{A}]_\mathrm{M}^\mathrm{TOT}, \qquad (S6)$$

$$[\mathrm{ADP}]_\mathrm{C} + [\mathrm{ATP}]_\mathrm{C} = [\mathrm{A}]_\mathrm{C}^\mathrm{TOT}. \qquad (S7)$$

Kinetic expressions for various fluxes

Adapted from Magnus and Keizer [3], Eq. S8 accounts for the effects of Pyruvate dehydrogenase (PDH)-catalyzed reaction, glycolytic pathway (k_{GLY}), and the Krebs cycle (reduction of $[NAD^+]_M$ into NADH) [6].

$$V_{PDH} = k_{GLY} \frac{1}{q_1 + \frac{[\text{NADH}]_\text{M}}{[\text{NAD}^+]_\text{M}}} \frac{[\text{Ca}^{2+}]_\text{M}}{q_2 + [\text{Ca}^{2+}]_\text{M}}. \quad (S8)$$

Another important pathway effected by mitochondrial Ca^{2+} influx is the aspartate-glutamate carrier (AGC) - a part of the MAS NADH shuttle system, and is given as

$$V_{AGC} = V_{AGC,max} \frac{[\text{Ca}^{2+}]_\text{C}}{K_{AGC} + [\text{Ca}^{2+}]_\text{C}} \frac{q_2}{q_2 + [\text{Ca}^{2+}]_\text{M}} e^{p_4 \Delta\Psi}. \quad (S9)$$

The rate of $[NADH]_M$ oxidation in the electron transport chain (ETC) and the coupled extrusion of protons from mitochondria are combined into one equation, i.e.

$$V_O = k_o \frac{[\text{NADH}]_\text{M}}{q_3 + [\text{NADH}]_\text{M}} \left(1 + e^{\frac{\Delta\Psi - q_4}{q_5}}\right)^{-1}. \quad (S10)$$

Where the exponential factor contains the dependency on both $\Delta\Psi$ and the proton (extrusion from mitochondria through electron transport cycle) gradient.

Flux due to adenine nucleotide translocator (also called ADP/ATP translocase or mitochondrial ADP/ATP carrier) exchanges free ATP with free ADP across the inner mitochondrial membrane and is given as

$$V_{ANT} = V_{ANT,max} \frac{1 - \frac{\alpha_C [\text{ATP}]_\text{C} [\text{ADP}]_\text{M}}{\alpha_M [\text{ADP}]_\text{C} [\text{ATP}]_\text{M}} e^{-\frac{F\Delta\Psi}{RT}}}{\left(1 + \alpha_C \frac{[\text{ATP}]_\text{C}}{[\text{ADP}]_\text{M}} e^{-0.5\frac{F\Delta\Psi}{RT}}\right)\left(1 + \frac{[\text{ADP}]_\text{M}}{\alpha_M [\text{ATP}]_\text{M}}\right)}. \quad (S11)$$

Here $[ATP]_C$ -$[ATP]_M$, $[ATP]_C$ –$[ADP]_M$, $[ADP]_C$-$[ATP]_M$, and $[ADP]_C$-$[ADP]_M$ represent 4 combinations of ligands and αs stand for the fact that only a fraction of nucleotides has access to the transporter. The membrane potential dependence of this flux is due to negatively charged ADP and ATP (3- and 4-, respectively).

The rate of ATP synthesis by F_1F_O-ATPase is given as

$$V_{FIFO} = V_{FIFO,max} \left(\frac{q_6}{q_6 + [\text{ATP}]_\text{M}}\right)\left(1 + e^{\frac{q_7 - \Delta\Psi}{q_8}}\right)^{-1}. \quad (S12)$$

The rate of ATP consumption (ATP hydrolysis) by ATP-consuming processes in the cytoplasm is also incorporated in the model.

$$V_{HYD} = K_{HYD} \frac{[\text{ATP}]_\text{C}}{[\text{ATP}]_\text{C} + K_h}. \quad (S13)$$

Finally, the Ohmic mitochondrial proton leak is given as

$$V_{H,leak} = q_9 \Delta\Psi + q_{10}. \quad (S14)$$

The values and meaning of various parameters used are given in Table S1.

Extracting single channel gating parameters from dwell-time distributions

For demonstration, we used the double-exponential behavior of open dwell-time histogram observed in [7]. Specifically, in the experiments in [7], MCU and the regulatory proteins were inserted into planar bilayer and electrophysiological recording of single channel activity was carried out. In one of these experiments (Figure S3B in [7]), the open dwell-time distribution could be fitted with double exponential with τ_1 = 12.06 ms and τ_2 = 161.92 ms. We used these time constants to generate experimental dwell-time distribution (green bars in Figure 6A, main text).

To fit the model to open dwell-time distribution, we follow the formalism developed in [8, 9]. We refer the interested reader to these papers for full details. Briefly, the mean transition time from state A to B can be obtained by multiplying the occupancy of state A with the inverse of probability flux between the two states. For example, if a channel in state A has *m* and *n* number of ligand (*L1* and *L2* respectively) molecules bound. The mean transition time from state A to state B where the channel has *m*+2 and *n* molecules of L1 and L2 bound respectively, is given as

$$T_{A\to B} = K_A L_1^m L_2^n \times \left[\frac{1}{j_{mn\to m+1n} L_1^{m+1} L_2^n} + \frac{1}{j_{m+1n\to m+2n} L_1^{m+2} L_2^n}\right].$$

Similarly, the mean transition time from state B to state A is given as

$$T_{B\to A} = K_B L_1^{m+2} L_2^n \times \left[\frac{1}{j_{mn\to m+1n} L_1^{m+1} L_2^n} + \frac{1}{j_{m+1n\to m+2n} L_1^{m+2} L_2^n}\right].$$

Where $K_A L_1^m L_2^n$ and $K_B L_1^{m+2} L_2^n$ are the occupancies of states A and B respectively, and *js* are the probability flux parameters that come from the fit.

The transition rates between the two states are simply the inverse of mean transition times. Thus following the above formalism, the transition rates between different states in the MCU model are given as follows.

$$R_{C_{00}\to C_{20}^{M1}} = \left[\frac{1}{j_{00\to 10}[Ca^{2+}]_C} + \frac{1}{j_{10\to 20}[Ca^{2+}]_C^2}\right]^{-1}, \quad \text{(S15A)}$$

$$R_{C_{20}^{M1}\to C_{00}} = \frac{1}{K_{C_{20}^{M1}}[Ca^{2+}]_C^2}\left[\frac{1}{j_{00\to 10}[Ca^{2+}]_C} + \frac{1}{j_{10\to 20}[Ca^{2+}]_C^2}\right]^{-1}, \quad \text{(S15B)}$$

$$R_{C_{00}\to C_{20}^{M2}} = \left[\frac{1}{\tilde{j}_{00\to 10}[Ca^{2+}]_C} + \frac{1}{\tilde{j}_{10\to 20}[Ca^{2+}]_C^2}\right]^{-1}, \quad \text{(S15C)}$$

$$R_{C_{20}^{M2}\to C_{00}} = \frac{1}{K_{C_{20}^{M2}}[Ca^{2+}]_C^2}\left[\frac{1}{\tilde{j}_{00\to 10}[Ca^{2+}]_C} + \frac{1}{\tilde{j}_{10\to 20}[Ca^{2+}]_C^2}\right]^{-1}, \quad \text{(S15D)}$$

$$R_{C_{20}^{M1}\to C_{30}} = \frac{1}{K_{C_{20}^{M1}}[Ca^{2+}]_C^2}\left[\frac{1}{j_{20\to 30}[Ca^{2+}]_C^3}\right]^{-1}, \quad \text{(S15E)}$$

$$R_{C_{30}\to C_{20}^{M1}} = \frac{1}{K_{C_{30}}[Ca^{2+}]_C^3}\left[\frac{1}{j_{20\to 30}[Ca^{2+}]_C^3}\right]^{-1}, \quad \text{(S15F)}$$

$$R_{C_{20}^{M2}\to O_{40}} = \frac{1}{K_{C_{20}^{M2}}\,[Ca^{2+}]_C^2}\left[\frac{1}{\tilde{j}_{20\to 30}[Ca^{2+}]_C^3} + \frac{1}{\tilde{j}_{30\to 40}[Ca^{2+}]_C^4}\right]^{-1}, \quad \text{(S15G)}$$

$$R_{O_{40}\to C_{20}^{M1}} = \frac{1}{K_{O_{40}}\,[Ca^{2+}]_C^4}\left[\frac{1}{\tilde{j}_{20\to 30}[Ca^{2+}]_C^3} + \frac{1}{\tilde{j}_{30\to 40}[Ca^{2+}]_C^4}\right]^{-1}, \quad \text{(S15H)}$$

$$R_{C_{30}\to O_{40}} = \frac{1}{K_{C_{30}}\,[Ca^{2+}]_C^3}\left[\frac{1}{j_{30\to 40}[Ca^{2+}]_C^4}\right]^{-1}, \quad \text{(S15I)}$$

$$R_{O_{40}\to C_{30}} = \frac{1}{K_{O_{40}}\,[Ca^{2+}]_C^4}\left[\frac{1}{j_{30\to 40}[Ca^{2+}]_C^4}\right]^{-1}, \quad \text{(S15J)}$$

$$R_{C_{30}\to C_{31}} = \frac{1}{K_{C_{30}}\,[Ca^{2+}]_C^3}\left[\frac{1}{j_{30\to 31}[Ca^{2+}]_C^3\,[Ca^{2+}]_M}\right]^{-1}, \quad \text{(S15K)}$$

$$R_{C_{31}\to C_{30}} = \frac{1}{K_{C_{31}}\,[Ca^{2+}]_C^3\,[Ca^{2+}]_M}\left[\frac{1}{j_{30\to 31}[Ca^{2+}]_C^3\,[Ca^{2+}]_M}\right]^{-1}, \quad \text{(S15L)}$$

$$R_{C_{31}\to C_{41}} = \frac{1}{K_{C_{31}}\,[Ca^{2+}]_C^3\,[Ca^{2+}]_M}\left[\frac{1}{j_{31\to 41}[Ca^{2+}]_C^4\,[Ca^{2+}]_M}\right]^{-1}, \quad \text{(S15M)}$$

$$R_{C_{41}\to C_{31}} = \frac{1}{K_{C_{41}}\,[Ca^{2+}]_C^4\,[Ca^{2+}]_M}\left[\frac{1}{j_{31\to 41}[Ca^{2+}]_C^4\,[Ca^{2+}]_M}\right]^{-1}, \quad \text{(S15N)}$$

$$R_{C_{41}\to O_{40}} = \frac{1}{K_{C_{41}}\,[Ca^{2+}]_C^4\,[Ca^{2+}]_M}\left[\frac{1}{j_{40\to 41}[Ca^{2+}]_C^4\,[Ca^{2+}]_M}\right]^{-1}, \quad \text{(S15O)}$$

$$R_{O_{40}\to C_{41}} = \frac{1}{K_{O_{40}}\,[Ca^{2+}]_C^4}\left[\frac{1}{j_{40\to 41}[Ca^{2+}]_C^4\,[Ca^{2+}]_M}\right]^{-1}, \quad \text{(S15P)}$$

$$R_{C_{41}\to O_{44}} = \frac{1}{K_{C_{41}}[Ca^{2+}]_C^4\,[Ca^{2+}]_M}\left[\frac{1}{j_{41\to 42}[Ca^{2+}]_C^4\,[Ca^{2+}]_M^2} + \frac{1}{j_{42\to 43}[Ca^{2+}]_C^4\,[Ca^{2+}]_M^3} + \frac{1}{j_{43\to 44}[Ca^{2+}]_C^4\,[Ca^{2+}]_M^4}\right]^{-1}, \quad \text{(S15Q)}$$

$$R_{O_{44}\to C_{41}} = \frac{1}{K_{O_{44}}\,[Ca^{2+}]_C^4\,[Ca^{2+}]_M^4}\left[\frac{1}{j_{41\to 42}[Ca^{2+}]_C^4\,[Ca^{2+}]_M^2} + \frac{1}{j_{42\to 43}[Ca^{2+}]_C^4\,[Ca^{2+}]_M^3} + \frac{1}{j_{43\to 44}[Ca^{2+}]_C^4\,[Ca^{2+}]_M^4}\right]^{-1}. \quad \text{(S15R)}$$

The model fit to the data is shown by the dashed line in (Figure 6A, main text). The values of various probability flux parameters given by the fit and their units are given in Table S3. Next, we used the transition rates listed above (Eqs. S15A-S15R) to stochastically simulate the gating of single MCU channel at different $[Ca^{2+}]_C$ and $[Ca^{2+}]_M$ values using the method previously developed [10-12] (Figure 6B, C main text).

Supplementary References

[1] B. Wacquier, L. Combettes, G.T.V. Nhieu, G. Dupont, Interplay Between Intracellular Ca2+ Oscillations and Ca2+-stimulated Mitochondrial Metabolism, Sci Rep-Uk, 6 (2016).
[2] G. Magnus, J. Keizer, Minimal model of beta-cell mitochondrial Ca2+ handling, Am J Physiol-Cell Ph, 273 (1997) C717-C733.
[3] G. Magnus, J. Keizer, Model of beta-cell mitochondrial calcium handling and electrical activity. II. Mitochondrial variables, Am J Physiol-Cell Ph, 274 (1998) C1174-C1184.
[4] G. Magnus, J. Keizer, Model of beta-cell mitochondrial calcium handling and electrical activity. I. Cytoplasmic variables, Am J Physiol-Cell Ph, 274 (1998) C1158-C1173.
[5] R. Bertram, M.G. Pedersen, D.S. Luciani, A. Sherman, A simplified model for mitochondrial ATP production, J Theor Biol, 243 (2006) 575-586.
[6] G.T. Hanson, R. Aggeler, D. Oglesbee, M. Cannon, R.A. Capaldi, R.Y. Tsien, S.J. Remington, Investigating mitochondrial redox potential with redox-sensitive green fluorescent protein indicators, J Biol Chem, 279 (2004) 13044-13053.
[7] M. Patron, V. Checchetto, A. Raffaello, E. Teardo, D.V. Reane, M. Mantoan, V. Granatiero, I. Szabò, D. De Stefani, R. Rizzuto, MICU1 and MICU2 finely tune the mitochondrial Ca2+ uniporter by exerting opposite effects on MCU activity, Molecular cell, 53 (2014) 726-737.
[8] G. Ullah, D.-O.D. Mak, J.E. Pearson, A data-driven model of a modal gated ion channel: The inositol 1, 4, 5-trisphosphate receptor in insect Sf9 cells, The Journal of general physiology, 140 (2012) 159-173.
[9] G. Ullah, W.J. Bruno, J.E. Pearson, Simplification of reversible Markov chains by removal of states with low equilibrium occupancy, Journal of theoretical biology, 311 (2012) 117-129.
[10] G. Ullah, P. Jung, Modeling the statistics of elementary calcium release events, Biophysical journal, 90 (2006) 3485-3495.
[11] D.-O.D. Mak, K.-H. Cheung, P. Toglia, J.K. Foskett, G. Ullah, Analyzing and quantifying the gain-of-function enhancement of IP3 receptor gating by familial Alzheimer's disease-causing mutants in Presenilins, PLoS computational biology, 11 (2015).
[12] G. Ullah, I. Parker, D.-O.D. Mak, J.E. Pearson, Multi-scale data-driven modeling and observation of calcium puffs, Cell calcium, 52 (2012) 152-160.
[13] R. Payne, H. Hoff, A. Roskowski, J.K. Foskett, MICU2 restricts spatial crosstalk between InsP3R and MCU channels by regulating threshold and gain of MICU1-mediated inhibition and activation of MCU, Cell reports, 21 (2017) 3141-3154.
[14] H. Vais, K. Mallilankaraman, D.-O.D. Mak, H. Hoff, R. Payne, J.E. Tanis, J.K. Foskett, EMRE is a matrix Ca2+ sensor that governs gatekeeping of the mitochondrial Ca2+ uniporter, Cell reports, 14 (2016) 403-410.
[15] M. Patron, V. Checchetto, A. Raffaello, E. Teardo, D. Vecellio Reane, M. Mantoan, V. Granatiero, I. Szabo, D. De Stefani, R. Rizzuto, MICU1 and MICU2 finely tune the mitochondrial Ca2+ uniporter by exerting opposite effects on MCU activity, Mol Cell, 53 (2014) 726-737.

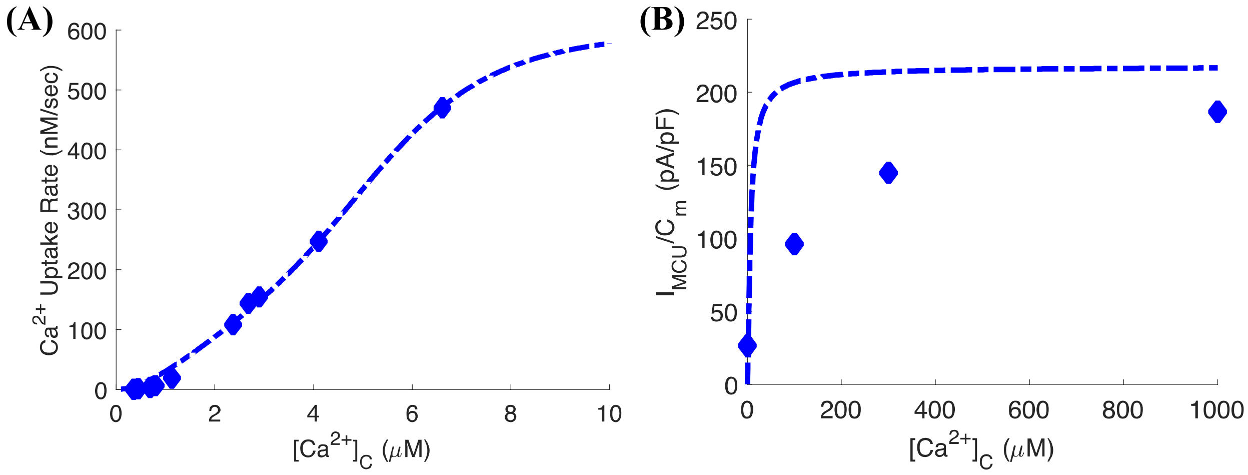

Figure S1: Using a different set of parameters improves the model fit to the MCU Ca^{2+} uptake rate observed in MICU2 KO cell cultures (A) but deteriorates the fit to MCU current density as a function of $[Ca^{2+}]_C$ at $[Ca^{2+}]_M = 0.4$ μM (B) in MICU2 KO mitoplasts. The occupancy parameters used are; $K_{C_{20}^{M1}} = 9.45876 \times 10^3/\mu M^2$, $K_{C_{20}^{M2}} = 6.10726397 \times 10^4/\mu M^2$, $K_{C_{30}} = 4.028223 \times 10^2/\mu M^3$, $K_{O_{40}} = 1.10819/\mu M^4$, $K_{C_{31}} = 2.0408 \times 10^4/\mu M^4$, $K_{C_{41}} = 1.144274 \times 10^{-4}/\mu M^5$, $K_{O_{44}} = 15.60478//\mu M^8$. Experimental data shown for comparison is from [13] (A) and [14] (B).

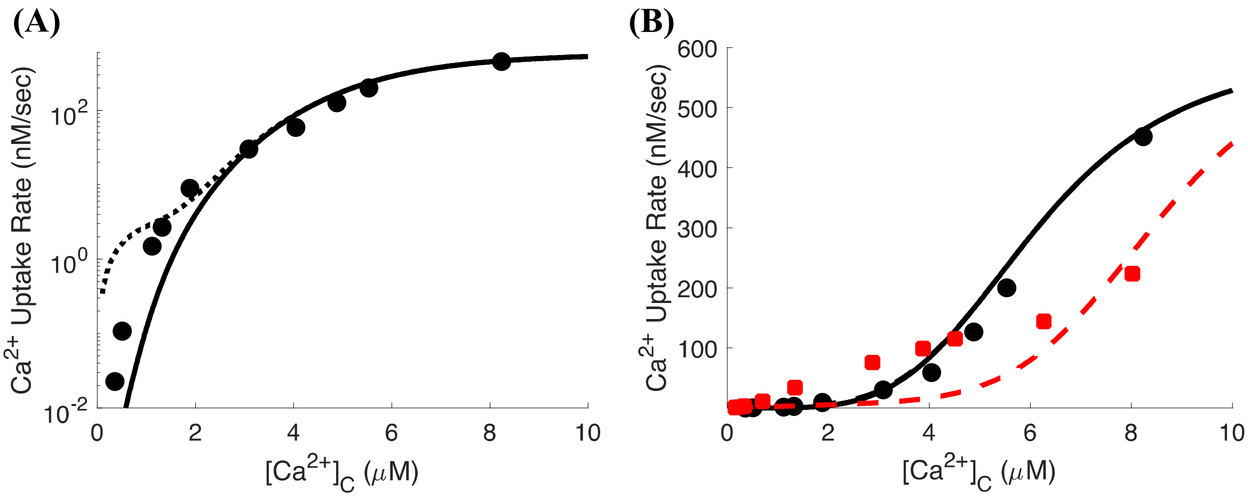

Figure S2: Considering the state with two and one Ca^{2+} bound to MICU1 and MICU2 respectively to be an open state results in higher Ca^{2+} uptake rate at low $[Ca^{2+}]_C$ when compared to observed values in WT cell cultures (A) and significantly deteriorates the overall fit to the observed uptake rate in MICU1 KO cell cultures (B). Experimental data shown for comparison is from [13].

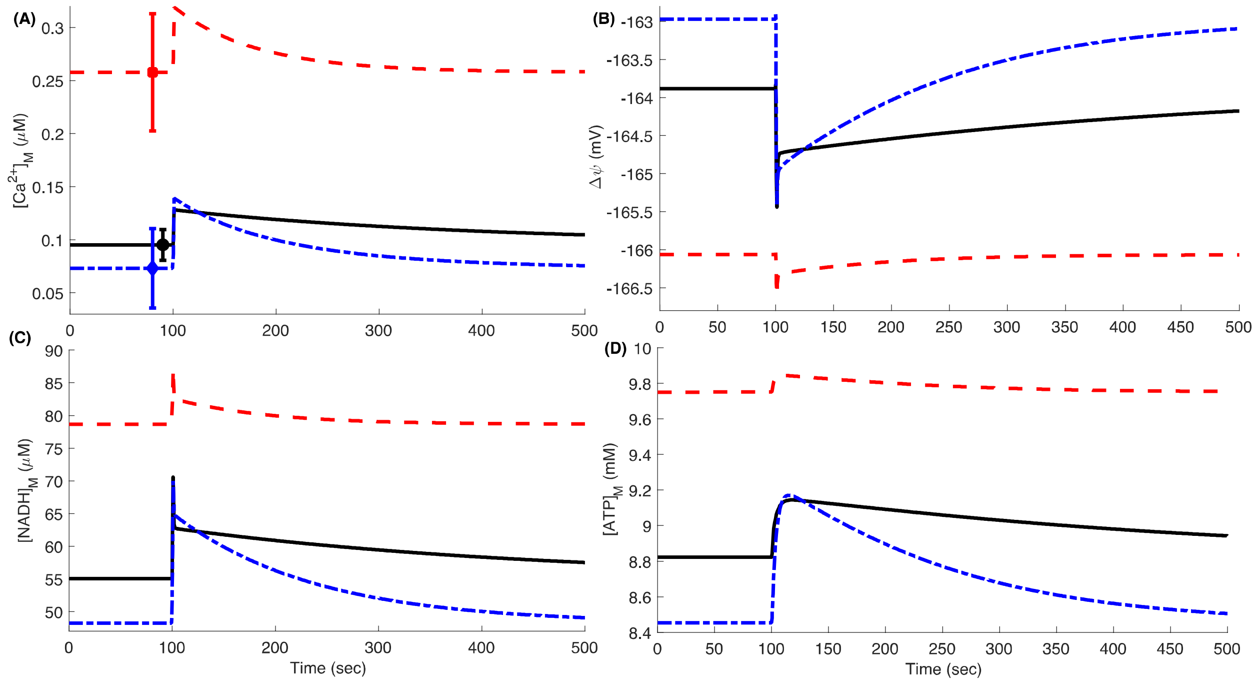

Figure S3: Changes in mitochondrial variables in response to a step-like increase in $[Ca^{2+}]_C$ at the whole-cell level. $[Ca^{2+}]_C$ is increased from its resting value of 0.1 μM to 1.5 μM for 1 sec starting at 100 sec into the simulation. Time trace of $[Ca^{2+}]_M$ (A), Δψ (B), $[NADH]_M$ (C), and $[ATP]_M$ (D). The solid, dashed, and dashed-dotted line corresponds to WT, MICU1 KO, and MICU2 KO cells respectively. The symbols in panel (A) represent the observed resting $[Ca^{2+}]_M$ in WT (sphere), MICU1 KO (square), and MICU2 KO (diamond) cells and the error bars represent $[Ca^{2+}]_M$ ± SEM (n = 5 for WT, MICU1 KO, and n = 8 for MICU2 KO conditions). Parameters used in these simulations are the same as given in the text except that the mitochondrial Ca^{2+} buffering capacity f_m is set to 0.09. $V_{NaCa,max}$ given by the model are 0. 958, 2.144, and 3.03 nM/s for WT, MICU1 KO, and MICU2 KO cells respectively. Experimental data shown for comparison is from [13] (A).

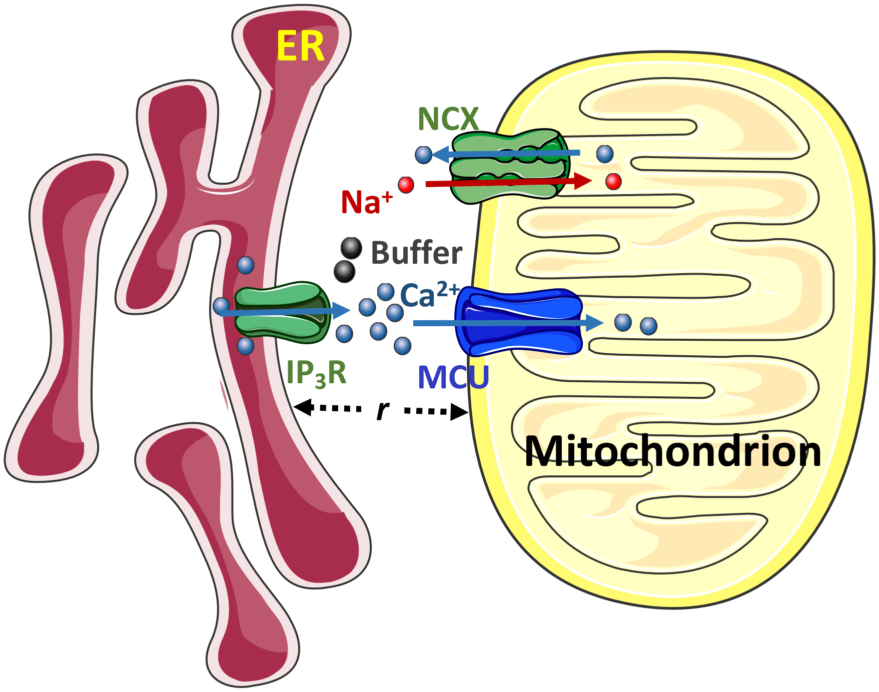

Figure S4: Schematic of the mitochondrial Ca^{2+} uptake model at the single mitochondrion level. Ca^{2+} released from the ER through IP_3R diffuses in the microdomain between the ER and mitochondrion, which is taken up by mitochondrion through MCU or buffered by Ca^{2+} binding proteins. Ca^{2+} is released back to the microdomain through Na^+/Ca^{2+} exchanger that extrudes one Ca^{2+} in exchange for three Na^+.

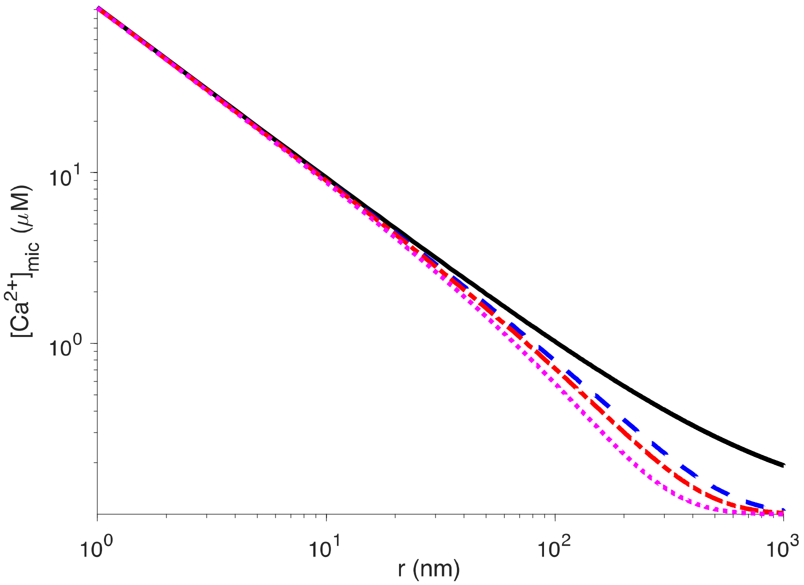

Figure S5: Ca^{2+} concentration in the microdomain ($[Ca^{2+}]_{mic}$) due to the opening of a single IP_3R as a function of distance from the channel at $B_T = 0$ (solid line), 100 (dashed line), 500 (dashed-dotted line), and 1000 μM (dotted line).

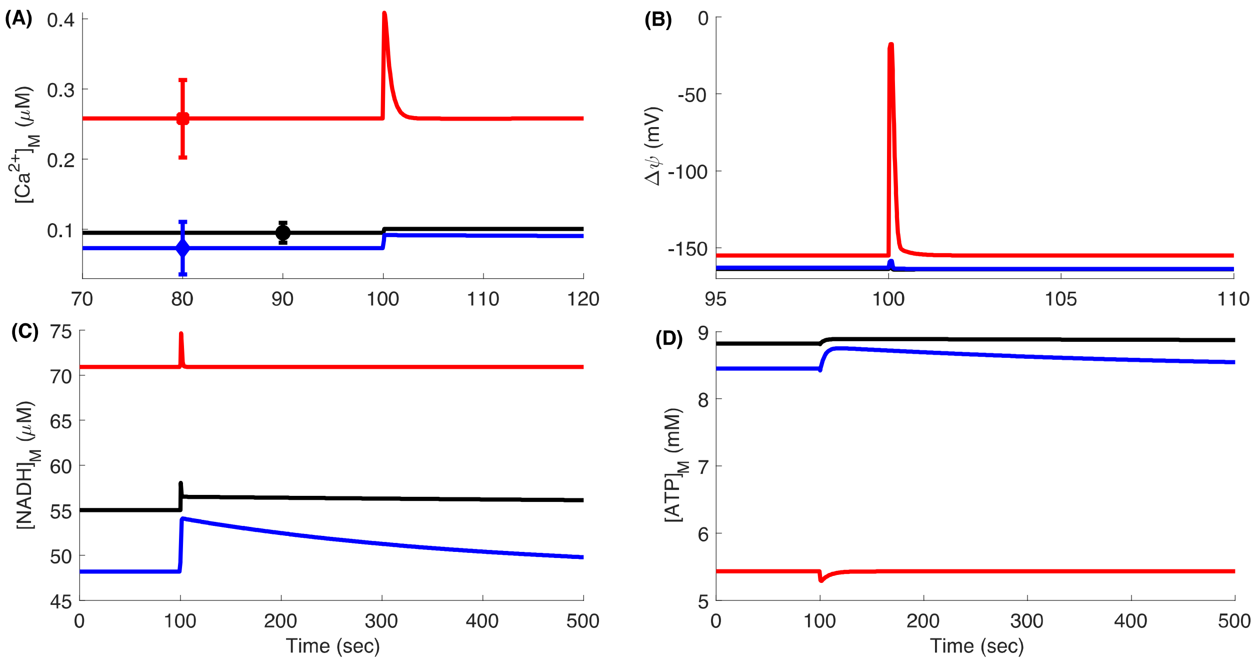

Figure S6: Changes in mitochondrial variables in response to the opening of a single IP_3R channel at the single mitoplast level. A single IP_3R residing in the ER membrane is allowed to open for 100 ms starting at 100 sec into the simulations. Time-traces of $[Ca^{2+}]_M$ (A), Δψ (B), $[NADH]_M$ (C), and $[ATP]_M$ (D) in WT (black lines), MICU1 KO (red lines), and MICU2 KO (blue lines) cells respectively. The symbols in panel (A) represent the observed resting $[Ca^{2+}]_M$ in WT (sphere), MICU1 KO (square), and MICU2 KO (diamond) cells and the error bars represent $[Ca^{2+}]_M \pm$ SEM (n = 5 for WT, MICU1 KO, and n = 8 for MICU2 KO conditions). Parameters used in simulations are the same as given in the text. The width of the microdomain in these simulations is 24 nm. The value of $V_{NaCa,max}$ given by the model is 1.112, 1838.6, and 35.9675 μM/s in WT, MICU1 KO, and MICU2 KO cells respectively. Experimental data shown for comparison is from [13] (A).

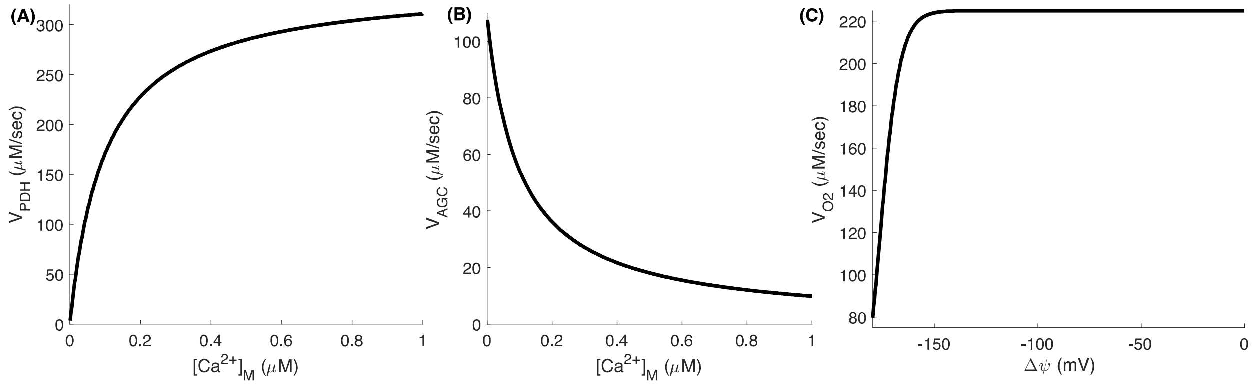

Figure S7: The behavior of the three reactions controlling $[NADH]_M$. Rates of pyruvate dehydrogenase-catalyzed reaction (A), malate-aspartate shuttle (B), and NADH oxidation/proton extrusion (C) as we change $[Ca^{2+}]_M$ or $\Delta\psi$ in the model.

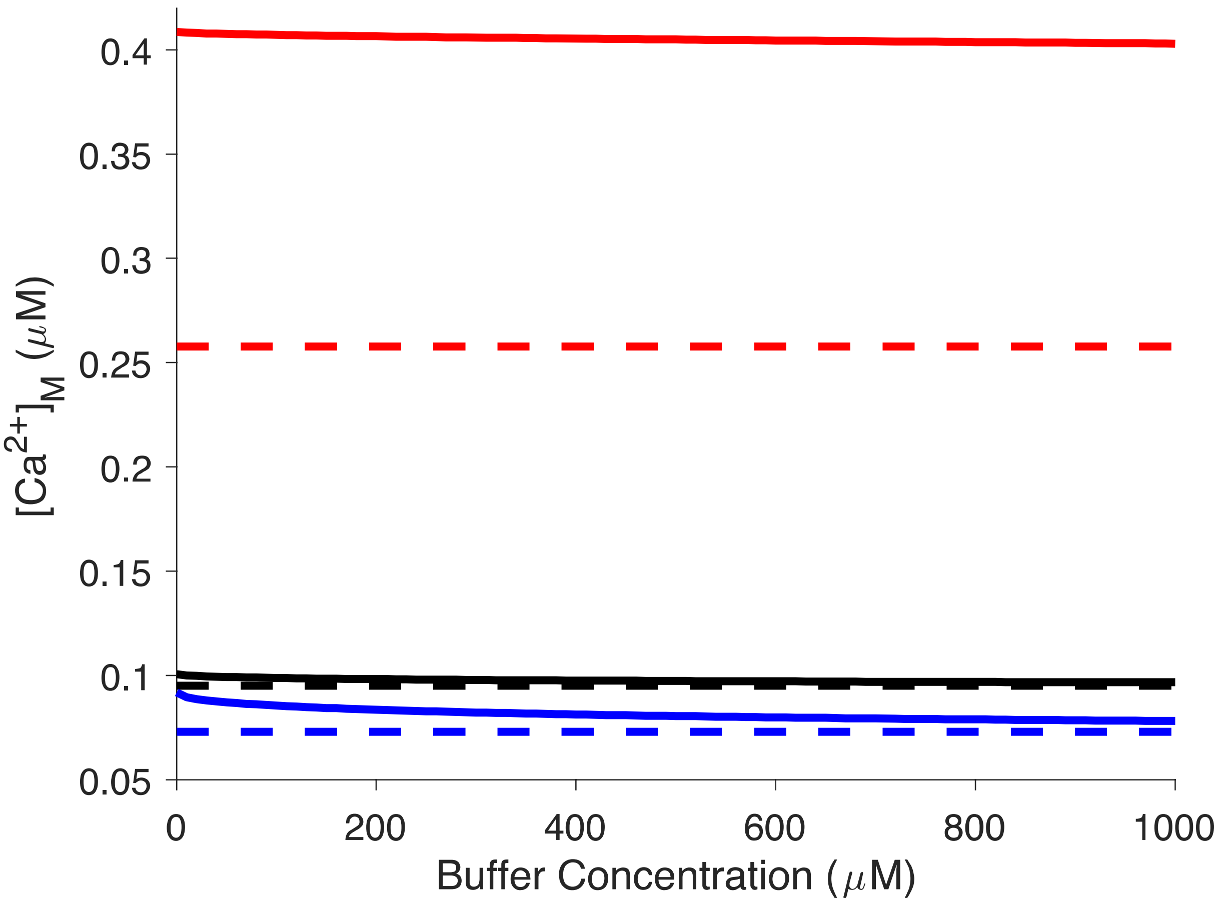

Figure S8: Changes in $[Ca^{2+}]_M$ in response to the opening of a single IP_3R channel at the single mitochondrion level as we vary the concentration of Ca^{2+} buffer in the cytoplasm. A single IP_3R residing in the ER membrane is allowed to open for 100 ms starting at 100 sec into the simulations and the resting (dotted line) and maximum (solid line) values of $[Ca^{2+}]_M$ are recorded in WT (black lines), MICU1 KO (red lines), and MICU2 KO (blue lines) cells. A 24 nm wide microdomain is considered in these simulations.

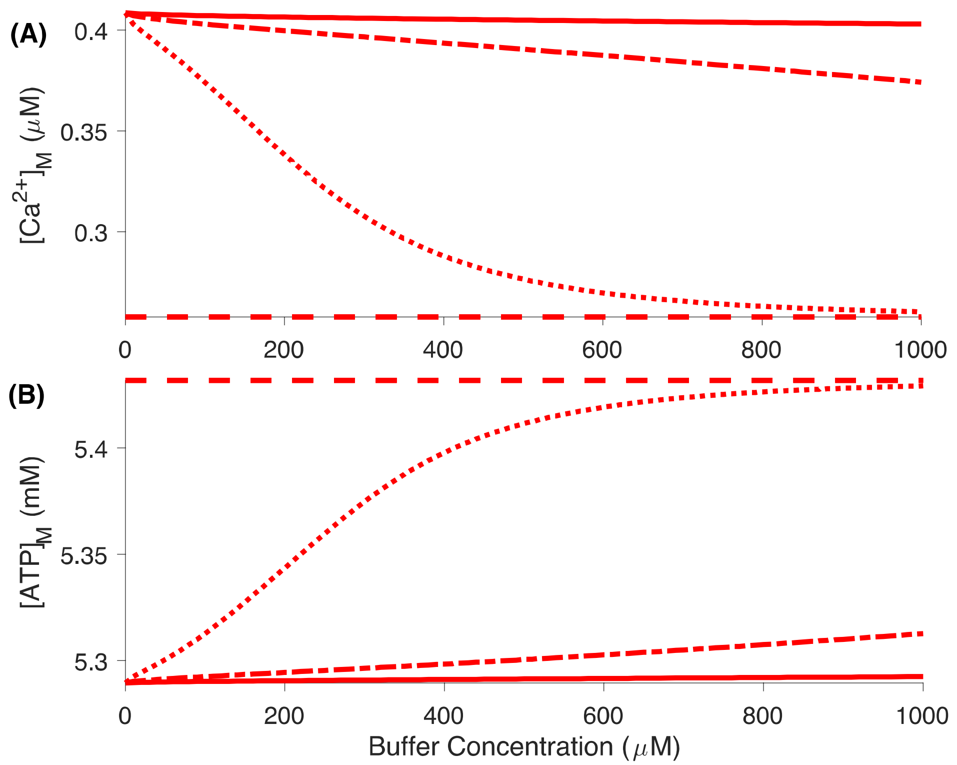

Figure S9: Changes in $[Ca^{2+}]_M$ (A) and $[ATP]_M$ (B) in response to the opening of a single IP_3R channel at the single mitochondrion level as we vary the concentration of Ca^{2+} buffer in the cytoplasm in MICU1 KO cells. A single IP_3R residing in the ER membrane is allowed to open for 100 ms starting at 100 sec into the simulations and the resting and maximum values of $[Ca^{2+}]_M$ are recorded using buffers with different kinetics. The optimum value of $[Ca^{2+}]_M$ (A) and $[ATP]_M$ (B) after IP_3R opens (note that $[ATP]_M$ decreases after IP_3R opens in case of MICU1 KO mitoplast) under control buffer (that described in the main text and used in Figure S8) (solid lines), a buffer with kinetics ten times faster than control (dashed-dotted lines), and a buffer with kinetics hundred times faster than control (dotted lines). The resting value of $[Ca^{2+}]_M$ and $[ATP]_M$ do not change as we change the kinetics of the buffer (dashed lines). A 24 nm wide microdomain is considered in these simulations.

Parameter	Definition	Value
a_1	Scaling factor between NADH consumption and change in membrane voltage	20
a_2	Scaling factor between ATP production by ATPase and change in membrane voltage	3.43
α_C	Factor taking cytosolic ADP and ATP buffering into account	0.111
α_M	Factor taking mitochondrial ADP and ATP buffering into account	0.139
A_C^{tot}	Total concentration of cytosolic adenine nucleotides	2500 µM
A_M^{tot}	Total concentration of mitochondrial adenine nucleotides	15000 µM
C_{mito}	Mitochondrial inner membrane capacitance divided by Faraday's constant	1.8 µM.mV^{-1}
δ	Mitochondrial to cytosolic volume ratio	varies
f_m	Fraction of free over buffer-bound Ca^{2+} in mitochondria	varies
K_{AGC}	Dissociation constant of Ca^{2+} from AGC	0.14 µM
k_{GLY}	Velocity of glycolysis (empirical)	450 µM.s^{-1}
K_h	Michaelis-Menten constant for ATP hydrolysis	1000 µM
k_{HYD}	Maximal rate of ATP hydrolysis	100 µM.s^{-1}
k_o	Rate constant of NADH oxidation by ETC	600 µM.s^{-1}
$[NAD]_M^{tot}$	Total concentration of mitochondrial pyridine nucleotides	250 µM
p_4	Voltage dependence coefficient of AGC activity	0.01 mV^{-1}
q_1	Michaelis-Menten-like constant for NAD^+ consumption by the Krebs cycle	1
q_2	$S_{0.5}$ value for activation the Krebs cycle by Ca^{2+}	0.1 µM
	$S_{0.5}$ value for indirect inhibition of the AGC by cytosolic Ca^{2+}	0.1 µM
q_3	Michaelis-Menten constant for NADH consumption by the ETC	100 µM
q_4	Voltage dependence coefficient 1 of ETC activity	177 mV
q_5	Voltage dependence coefficient 2 of ETC activity	5 mV
q_6	Inhibition constant of ATPase activity by ATP	10000 µM
q_7	Voltage dependence coefficient of ATPase activity	190 mV
q_8	Voltage dependence coefficient of ATPase activity	8.5 mV
q_9	Voltage dependence of the proton leak	2 µM.s^{-1}. mV^{-1}
q_{10}	Rate constant of the voltage-independent proton leak	-30 µM.s^{-1}
$V_{ANT,max}$	Maximum rate of adenine nucleotide translocator	5000 µM.s^{-1}
$V_{AGC,max}$	Maximum rate of NADH production via malate-aspartate shuttle	25 µM.s^{-1}
$V_{FIFO,max}$	Maximum rate of F1FO ATPase	35000 µM.s^{-1}

Table S1. Meaning and values of various parameters used in the bioenergetics model.

Parameter	WT Cells	MICU1 KO Cells	MICU2 KO Cells
$K_{C_{20}^{M1}}$	9.45876×10^3 / μM^2	4.23402×10^3 / μM^2	4.99195×10^3 / μM^2
$K_{C_{20}^{M2}}$	6.10727×10^4 / μM^2	3.29724×10^4 / μM^2	4.98561×10^4 / μM^2
$K_{C_{30}}$	0.17769 / μM^3	2.47972×10^5/ μM^3	7.36347×10^2/ μM^3
$K_{C_{31}}$	2.04082×10^4 / μM^4	0 / μM^4	0 / μM^4
$K_{C_{41}}$	3.11575 / μM^5	0 / μM^5	0 / μM^5
$K_{O_{40}}$	2.14902 / μM^4	1.37294×10^4/ μM^4	9.45846 / μM^4
$K_{O_{44}}$	15.70201 / μM^8	0.95343 / μM^8	21.44413 / μM^8

Table S2: Values of various parameters given by the fit of the kinetic model for MCU function to the observed open probability of MCU as a function of $[Ca^{2+}]_C$ and $[Ca^{2+}]_M$.

Parameter	Value	Unit
$j_{00\to10}$	4.98 x 10^2	$[msec.\mu M]^{-1}$
$j_{10\to20}$	4.98x 10^2	$[msec.\mu M^2]^{-1}$
$\tilde{j}_{00\to10}$	4.98x 10^2	$[msec.\mu M]^{-1}$
$\tilde{j}_{10\to20}$	4.98x 10^2	$[msec.\mu M^2]^{-1}$
$j_{20\to30}$	4.98x 10^2	$[msec.\mu M^3]^{-1}$
$\tilde{j}_{20\to30}$	11.0x 10^{-2}	$[msec.\mu M^3]^{-1}$
$\tilde{j}_{30\to40}$	11.0x 10^{-2}	$[msec.\mu M^4]^{-1}$
$j_{30\to40}$	2.0x 10^{-4}	$[msec.\mu M^4]^{-1}$
$j_{30\to31}$	4.98x 10^2	$[msec.\mu M^4]^{-1}$
$j_{31\to41}$	4.98x 10^2	$[msec.\mu M^5]^{-1}$
$j_{40\to41}$	3.0x 10^{-4}	$[msec.\mu M^5]^{-1}$
$j_{41\to42}$	52.0x 10^{-2}	$[msec.\mu M^6]^{-1}$
$j_{42\to43}$	52.0x 10^{-2}	$[msec.\mu M^7]^{-1}$
$j_{43\to44}$	52.0x 10^{-2}	$[msec.\mu M^8]^{-1}$

Table S3: Values of various probability flux parameters given by the fitting the model to open dwell-time distribution generated using the time constants (τ_1 = 12.06 ms and τ_2 = 161.92 ms) observed in lipid bilayer experiments [15].
